# *Klebsiella pneumoniae *related community-acquired acute lower respiratory infections in Cambodia: Clinical characteristics and treatment

**DOI:** 10.1186/1471-2334-12-3

**Published:** 2012-01-10

**Authors:** Blandine Rammaert, Sophie Goyet, Julien Beauté, Sopheak Hem, Vantha Te, Patrich Lorn Try, Charles Mayaud, Laurence Borand, Philippe Buchy, Bertrand Guillard, Sirenda Vong

**Affiliations:** 1 Institut Pasteur in Cambodia, Réseau International des Instituts Pasteur, Phnom Penh, Cambodia; 2 Donkeo Provincial Hospital, Takeo, Cambodia; 3 Kampong Cham Provincial hospital, Kampong Cham, Cambodia; 4 Centre de pneumologie et réanimation respiratoire, Hôpital Tenon, Paris, France

**Keywords:** *Klebsiella pneumoniae*, Community-acquired, Pneumonia, Extended-spectrum betalactamases, Diabetes mellitus

## Abstract

**Background:**

In many Asian countries, *Klebsiella pneumoniae *(KP) is the second pathogen responsible for community-acquired pneumonia. Yet, very little is known about *KP *etiology in ALRI in Cambodia, a country that has one of the weakest medical infrastructures in the region. We present here the first clinico-radiological description of *KP *community-acquired ALRI in hospitalized Cambodian patients.

**Methods:**

Through ALRI surveillance in two provincial hospitals, *KP *was isolated from sputum and blood cultures, and identified by API20E gallery from patients ≥ 5 years-old with fever and respiratory symptoms onset ≤14 days. Antibiotics susceptibility testing was provided systematically to clinicians when bacteria were isolated. We collected patients' clinical, radiological and microbiological data and their outcome 3 months after discharge. We also compared *KP*-related with other bacteria-related ALRI to determine risk factors for *KP *infection.

**Results:**

From April 2007 to December 2009, 2315 ALRI patients ≥ 5 years-old were enrolled including 587 whose bacterial etiology could be assigned. Of these, 47 (8.0%) had *KP *infection; their median age was 55 years and 68.1% were females. Reported prior medication was high (42.5%). Patients' chest radiographs showed pneumonia (61.3% including 39% that were necrotizing), preexisting parenchyma lesions (29.5%) and pleural effusions alone (4.5%) and normal parenchyma (4.5%). Five patients had severe conditions on admission and one patient died during hospitalization. Of the 39 patients that were hospital discharged, 14 died including 12 within 1 month after discharge. Only 13 patients (28%) received an appropriate antibiotherapy. Extended-spectrum beta-lactamases (ESBL) - producing strains were found in 8 (17.0%) patients. Female gender (Odds ratio (OR) 2.1; *p *= 0.04) and diabetes mellitus (OR 3.1; *p *= 0.03) were independent risk factors for *KP*-related ALRI.

**Conclusions:**

*KP *ALRI in Cambodia has high fatality rate, are more frequently found in women, and should be considered in diabetic patients. The extremely high frequency of ESBL-producing strains in the study is alarming in the context of uncontrolled antibiotic consumption and in absence of microbiology capacity in most public-sector hospitals.

## Background

*Klebsiella pneumoniae *(*KP*) is a Gram negative bacillus often involved in severe community-acquired and nosocomial infections [1]. It has been proven to be an independent risk factor for mortality in severe community-acquired pneumonia (CAP) [2]. Its distribution as a CAP pathogen is uneven across the world with a highest incidence in developing countries and in Asia [3-5]. To explain these geographical patterns, hypotheses include differences in virulence of strains, host defences and the importance of antibiotic consumption in self-medication [6,7]. *KP *is naturally resistant aminopenicillins (i.e. ampicillin or amoxicillin), an antibiotic that is commonly used as the first line antibiotherapy in CAP in many developing countries. This raises serious concerns since a recent multicenter prospective study conducted in several Asian countries (not including Cambodia) showed *KP *accounted for 15.4% of the pathogens responsible for hospitalized CAP, just after *Streptococcus pneumoniae *[8]. Last, emerging resistances among Gram-negative bacilli such as extended-spectrum betalactamases (ESBL) are increasingly recognized as a major public health issue in both developed and developing countries [9,10].

In Cambodia - one of the poorest countries in the region with ill-equipped health care facilities - microbial identification is a challenge and antimicrobial resistance detection is poorly documented. This study aimed at better characterizing hospitalized patients with *KP-*related acute lower respiratory infections (ALRI) in Cambodia collecting clinical, radiological and microbiological data.

## Methods

Beginning in April 2007, we conducted surveillance of ALRI in two Cambodian provincial hospitals. Eligible patients (≥5 years-old) included those with ALRI defined by onset symptoms ≤14 days, a fever of ≥ 38°C (axillary temperature using digital thermometers) or a history of fever within the previous 3 days, recent cough, plus at least one respiratory symptom (i.e. dyspnea, chest pain or crackles on lung auscultation). Patients with known tuberculosis (TB) or known acquired immunodeficiency were not included. For each participant, hospital physicians filled out a standardized case report form including information on patient's medical history, clinical features, treatment, laboratory and radiological findings and status at discharge. Medical records and chest X-rays were retrospectively reviewed by an expert pulmonologist. A home visit was conducted at least 3 months following hospital discharge to determine the outcome of the *KP-*infected patients. The surveillance project was approved by the National Ethical Committee. All patients and parents of <18 year-old patients who participated provided written informed consent.

### Specimens' collection and microbiology

Blood, non-induced sputum, and nasopharyngeal swabs were collected in each participant once within 48 h after hospital presentation for direct examination, cultures and molecular diagnostic. Sputum and blood (10 mL in Hemoline Diphasic Performance bottle (bioMérieux, Marcy l'Etoile, France) for adult and Isolator 1.5 (Oxoid, Basingstoke, England) (1.5 mL for children) were sent to Institut Pasteur in Cambodia to perform Gram stain and culture. Only good-quality sputum specimens were submitted for bacterial cultures using Murray-Washington's score (>25 polynuclear leukocytes and <25 squamous epithelial cells per low-power field) [11]. Sputum specimens were inoculated on colistin-nalidixic acid agar, cystine-lactose-electrolyte-deficient agar, chocolate polyvitex agar with bacitracin and Ashdown agar. Bacterial isolates were identified by API gallery (BioMérieux). Antibiotic susceptibility testing was performed by disk-diffusion method on Mueller-Hinton agar plates (Bio-Rad, Marnes-la-Coquette, France). ESBL production was detected by using the double-disk synergy test (clavulanic acid and cefotaxime, ceftazidime, cefepime and aztreonam) performed on Mueller-Hinton media.

Complete microbiology results were returned to the patient's hospital clinicians as soon as they were available. Microscopy for acid fast bacilli detection was systematically performed at the hospital laboratory, and TB culture where indicated in the guidelines of the national TB program.

### Definitions

*KP*-infected cases were defined by identification of *KP *in either blood or sputum culture. A significant growth of *KP *was defined as greater than 10^7 ^organisms per mL of original sputum.

A severe case was defined by the presence of at least two of the following criteria: systolic blood pressure (BP) < 90 mmHg, cardiac frequency ≥120 bpm, respiratory rate ≥30/mn, oxygen saturation <90%, temperature <35°C or ≥40°C. Severity definition was adapted to the Cambodian setting from PSI and CRB65 scores [12]. Since no patient was taking anti-diabetic drug and fasting plasma glucose was not always available, we defined as diabetic every patient with glycemia >7.8 μmol/L or with a medical history of diabetes mellitus [13].

Renal impairment was defined as a serum creatinine >150 μmol/L and liver disease by a history of chronic liver involvement. Cardiovascular disease was defined by a history of high blood pressure or cardiomegaly on chest X-ray or known cardiopathy. Having TB was defined by a positive AFB smear, as 97% of them were linked to a positive culture for *Mycobacterium tuberculosis *in Cambodian hospitalized patients (unpublished data). A necrotizing pneumonia was defined by the presence of either cavity or abscess, unique or multiple. We created a separate preexisting lung lesions diagnostic group as these lesions may alter or disguise the appearance of a pneumonic infiltrate. The ALRI with normal radiography group consisted of patients presenting with symptoms of acute bronchitis and normal lung parenchyma radiographs [14,15]. Expert pulmonologists retrospectively interpreted radiographs unaware of the microbiology diagnosis.

### Statistical analyses

We analyzed variables associated with mortality among patients with *KP *infection. In addition, we compared *KP-infected *cases with ALRI cases infected with *Haemophilus influenzae *and *Streptococcus pneumoniae *(*H&S*), the two main pyogenic bacteria identified in patients in both hospitals to determine clinical/radiological characteristics associated with *KP *infection. Variables that were tested by univariate analysis included age, gender, comorbidities, prior treatment to admission, hemoptysis, chest pain, severity on admission, pneumonia, necrotizing pneumonia, uncomplicated pneumonia, pleurisy, preexisting pulmonary lesions and TB co-infection. For mortality-associated risk factors, the variable "receiving an appropriate antibiotherapy" was added into the analyses. Variables for which a *p*-value in stratified analysis was ≤0.2, were further analyzed using a stepwise forward logistic regression model. Statistical analyses were performed using STATA version 9.2 (Statacorp, College Station, TX, USA). Odds ratio (OR) and 95% confidence interval (CI) were calculated for all variables significantly associated with *KP *infection or mortality.

Continuous variables were presented with their median and inter quartile range (IQR) or mean and minimum/maximum ranges. Variables were compared across groups using Wilcoxon-Mann-Whitney test for continuous variables, and the Chi-square or Fisher's exact test for categorical variables. For all analyses, statistical significance was defined at *P *< 0.05.

## Results

### Patients characteristics

From April 2007 to December 2009, 2315 ALRI patients ≥5 years-old were enrolled including 587 whose bacterial etiology could be assigned. Of these, 47 (8.0%) had *KP *infection. The median age of *KP*-infected cases was 55 years (range 25-79). Women were predominant (n = 32; 68.1%); of which 72% aged >45 years. Main comorbidities or known risk factors included chronic lung disease (n = 13), smoker (n = 10), cardiovascular disease (n = 7), diabetes mellitus (n = 7), alcoholism (n = 6), renal impairment (n = 3), liver disease (n = 1). Treatment prior to hospitalization was reported in 20 patients (42.5%), of which 5 reported the use of antibiotics. Reports of cigarette smoking and excessive alcohol drinking (≥1 glass of rice wine or few beer cans per day) were significantly more frequent in males than females (6/14 versus 0/29; *p *< 0.001). Distribution of other comorbidities did not significantly differ between genders or age groups.

### Clinical, biological and radiological features on admission

Onset of symptoms occurred on average within 9 days before presentation (range 1-14 days).

On admission, patients presented with fever >38°C (n = 30; 63.8%), chest pain (n = 28; 59.6%), cough (n = 26; 55.3%), and/or hemoptysis (n = 11; 23.4%). Five (10.6%) patients had at least 2 severity criteria of which none had hemoptysis. Interpretation of the 44 chest radiographs that were available yielded 27 (61.3%) pneumonia (i.e. 2 associated with pleural effusion and 14 with cavitations), 13 (29.5%) preexisting pulmonary lesions (bronchiectasis and/or infectious sequelae with cavitation, fibrosis, retraction, pleural thickening), 2 (4.5%) pleural effusion and 2 with normal parenchyma. For the latter group, both radiographies showed a thoracic hyperinflation, suggestive of a chronic obstructive pulmonary disease. Of the 10 patients who had hemoptysis and available chest X-rays, 4 had preexisting lung lesions and 6 had pneumonia including 5 with cavitations. Of note, AFB were found on direct sputum examination in only 2 cases. Among the 5 severe cases, 3 had pneumonia without cavities, 1 had pleural effusion and 1 had preexisting lung lesions. Females had significantly more radiological evidence of pneumonia than males (55.6% vs. 44.4% respectively; *p *= 0.02).

### Microbiological data

*KP *grew from 46 (97.8%) sputum cultures and 1 blood culture. No patients had both positive sputum and blood cultures. In 5 cases, other bacteria (*S. pneumoniae*, n = 3; *H influenza*, n = 1, *Staphylococcus aureus*, n = 1) grew together with *KP*. AFB was also identified in six (12.8%) patients. Viruses (respiratory syncytial virus, n = 7; influenza B virus, n = 2; human rhinovirus, n = 2, including one rhinovirus and respiratory syncytial virus co-infection) was found in 21.3% (n = 10/47) of the patients. Bacterial or viral coinfections were not associated with severity. Resistance to cotrimoxazole was present in 32.3% (n = 10) of the strains and resistance to ciprofloxacin was detected in only one strain. ESBL production was detected in 17.0% (n = 8) of the strains cultured from sputum specimens. All patients infected with ESBL-producing strains were women. One patient had a medical history of diabetes mellitus, one had a cardiopathy and another one had pulmonary TB sequelae. Comorbidities distribution was not significantly different in patients with and without ESBL-producing *KP*. ESBL production was frequently associated with gentamicin resistance (50%; n = 4/8) and in most cases with susceptibility to ciprofloxacin (n = 7/8) and fosfomycine (n = 8). Susceptibility testing of the 39 non-ESBL producing *KP *strains were as follows: amoxicillin/clavulanic acid, 94.7% (37/39), cefotaxime, 97.4% (38/39), cefepime, 96.7% (29/30), ceftazidime, 97.4% (38/39), ticarcilline, 2.5% (1/39), imipenem 97.4% (38/39), nalidixic acid, 83.3% (25/30), ciprofloxacine, 92.3 (36/39), gentamicin, 89.7% (35/39), amikacin, 97.4% (38/39), cotrimoxazole, 61.3% (19/31).

### Comparison between KP and H&S infected patients

As 4 patients were co-infected with *KP *and *H&S*, we compared the 43 *KP *to the 160 *H&S *infected patients (122 *H. influenzae *and 58 *S. pneumoniae*) (Table 1). There was a significantly higher proportion of females (67.4 vs. 50.0%; *p *= 0.04) in the *KP *group compared with the *H&S *group. Of all reported co-morbidities, only diabetes mellitus was more frequently associated with *KP*-infected patients than *H&S*-infected patients (16.3% vs 6.0%; *p *= 0.02). Biological results did not differ between *KP *and *H&S *infected patients (Table 1).

**Table 1:** Characteristics on admission of *K. pneumoniae *infected patients compared to patients with *H. influenzae/S pneumoniae *ALRI aged ≥5 years in univariate analysis

	Patients with *K. pneumoniae *ALRI (n = 43)	Patients with *H. influenzae *and *S. pneumoniae *ALRI (n = 160)	*P-value*
Age, median years (Inter Quartile Range [IQR])	55 (45-72)	57 (42-65)	*0.8*

Male gender (%)	14 (32.6)	80 (50.0)	*0.04*

Co-morbidities (%)			

Smoking history	10 (23.3)	50 (31.2)	*0.3*

Cardiovascular disease	6 (13.9)	17 (10.6)	*0.5*

Diabetes mellitus	7 (16.3)	10 (6.2)	*0.03*

Alcoholism	6 (13.9)	35 (21.9)	*0.2*

Renal impairment	3 (7.0)	10 (6.2)	*0.8*

Liver disease	1 (2.3)	1 (0.6)	*0.3*

Prior treatment	19 (44.2)	92 (57.5)	*0.08*

Prior antibiotic	5 (11.6)	7 (4.4)	*0.08*

Clinical data			

Chest pain	24 (55.8)	106 (66.2)	*0.1*

Hemoptysis	10 (23.2)	23 (14.4)	*0.1*

Severity criteria	5 (11.6)	8 (5.0)	*0.1*

Laboratory results			

Leucocytes, median 10^9^/L (IQR)	8.5 (6.7 - 10.8)	8.3 (6.2 - 10.6)	*0.6*

AST*, median U/L (IQR)	29 (25-42)	27.5 (21-38)	*0.1*

ALT*, median U/L (IQR)	30 (21-32)	26 (20-34)	*0.4*

Blood creatinine μmol/L (IQR)	88.4 (77.8 - 108.7)	88.4 (76.0 - 99.0)	*0.6*

**NOTE**. Liver enzymes: AST (Aspartate transaminase) and ALT (Alanine transaminase)

There was no difference between radiological findings in both groups as shown in Table 2. Based on multivariate analysis, female gender (OR = 2.1; 95%CI [1.0-4.5]; *p *= 0.04) and diabetes mellitus (OR = 3.1; 95%CI [1.1-9.1]; *p *= 0.03) were independent risk factors for *KP*-related ALRI.

**Table 2:** Radiological data on admission for 40 *K. pneumoniae *compared to 146 *H. influenzae & S.pneumoniae *infected patients aged ≥5 years in univariate analysis

	Patients with *K. pneumoniae *ALRI N = 40 (%)	Patients with *H. influenzae *and *S. pneumoniae *ALRI N = 146 (%)	*P-value*
Pneumonia	25 (62.5)	68 (46.6)	*0.05*

Consolidation	16 (40.0)	51 (34.9)	*0.3*

Consolidation with cavitation	9 (22.5)	17 (11.6)	*0.07*

Consolidation with pleural effusion	2 (5.0)	6 (4.1)	*0.5*

Pleural effusion alone	1 (2.5)	0 (0.0)	*0.2*

Preexisting lung diseases	12 (30.0)	62 (42.5)	*0.1*

Normal parenchyma^a^	2 (5.0)	16 (10.9)	*0.2*

**NOTE**. Data are no. (%) of patients

^a^A chronic pulmonary disease cannot be excluded as chest computed tomography scan and pulmonary function tests were not available

### Treatment and outcome

In the *KP *group, 47 received antibiotics during their hospital stay, two patients were transferred to another ward or hospital and one left the hospital before being treated, probably because the family could not afford the hospitalization costs. Appropriate first line antimicrobial therapy (ceftriaxone, amoxicillin/clavulanic acid, cotrimoxazole or ciprofloxacin) was prescribed to only four patients (9.0%). In the remaining 40 patients, penicillin A was given (n = 39/40; 97.5%), in combination with gentamicin in 13 patients (33%) while one patient only received macrolides. This antimicrobial therapy was appropriately modified in nine (22.5%). Only 2 of 8 patients with ESBL-producing *KP *received an appropriate treatment and were considered as cured. For these patients, the treatment was adapted following antibiotic susceptibility. The overall length of stay at the hospital was on average 10.7 days (range 1-35) and longer for patients appropriately treated compared with that of patients without appropriate treatment (13.3 (range 3-21) vs 9.7 days (range 1-35); *p *= 0.01). On discharge, 59.6% (n = 28/47) of the patients were considered as cured by clinicians, one patient died during hospitalization and the outcome was unknown for 18 (38.3%) patients (14 were transferred to another ward or hospital and 4 escaped). Thirty-nine patients could be located for home visit. Of these, 14 (35.9%) died including 12 and two within 1 month and 6 weeks after discharge, respectively; none received an appropriate treatment. Twenty five (64.1%) were considered having recovered of which 13 received an appropriate treatment. Among the eight patients infected by ESBL-producing strains, three died within 1 month, two were not found and three were cured including two who received an appropriate antimicrobial therapy. As a result, the overall case fatality rate was 37.5% (15/40). Receiving an incorrect antimicrobial therapy was not a risk factor for mortality, although comorbidities, clinical and radiological features were not significantly different between survivors and non survivors (*p *= 0.12). Of the 12 patients who received at discharge incorrect treatment, none were considered severe. At the follow-up visit, 43.3% (13/30) of the patients who did not receive an appropriate treatment were alive.

## Discussion

This is the first clinico-radiological description of a substantial number of community-acquired *KP *ALRI in hospitalized patients in Cambodia. Our findings confirmed that *KP*-related respiratory infections carried poor prognosis with high fatality rates, longer hospital stays and was associated with older ages and co-morbidities. More importantly, *KP *pneumonia was frequently unsuspected and the initial antimicrobial treatment was often inappropriate. Yet, we found from this surveillance project that *KP *was the second pyogenic bacterium found among severe CAP in patients aged 5 years and older, and the 4th in infections on preexisting pulmonary lesions in Cambodia (Institut Pasteur's unpublished data). Moreover, the high frequency of ESBL-producing strains in *KP *infection was particularly worrisome. The ESBL resistance among community-acquired infections varied from 0% (Taiwan, South Korea, Malaysia) to 36.4% (India), but the incidence is not well studied in Cambodia or neighboring countries [16]. In a multicenter study in Asian countries determining CAP etiologies, antibiotic susceptibility testing was done for 36 strains of *KP *[8]. All but one were susceptible to ceftriaxone. Another international multicenter study conducted in Taiwan, Argentina, South Africa, Europe, United States, and Australia, showed an incidence of community-acquired ESBL-producing *KP *was of 3.5% (n = 7/202), all patients but one had a history of prior hospitalization [6]. In contrast, none of our patients with ESBL-producing strain reported previous hospitalization within the month prior to admission. In our cohort, 42.5% of the patients reported having taken treatment prior to hospitalization although the proportion of actual intake of antimicrobial drugs was not known. In the Cambodian context, it is believed however that this proportion may be high as patients have access to antibiotics and corticosteroids without medical prescription, through formal, informal and unregulated private health sectors [17]. Overuse and misuse (e.g. incorrect dosage) of antibiotics is a known risk factor for developing ESBL-producing strains [18]. Interestingly, a Cambodian study recently conducted in the capital city showed that up to 37% of the *E. coli *strains isolated from community acquired urinary tract infections carried ESBLs, of which all were CTX-M type [19].

Female gender and diabetes mellitus are independent risks factors associated with *KP *ALRI in Cambodia. Surprisingly, women accounted for more than two thirds (68.1%) of all cases. Published data report that community-acquired *KP *are associated with male gender, women accounting for 20% to 42% of the studied populations [1,6,20,21]. There was a trend to a higher antibiotic intake prior to hospitalization in women, whereas no data informed about health seeking behavior among women in Cambodia to confirm this tendency. Whereas age was not statistically different between men and women, 72% of women in our study were postmenopausal. Postmenopausal women are at risk to develop certain infections such as atypical mycobacterial infections [22]. In animals' models, the role of sex hormones in susceptibility to lung infections has been well demonstrated [23]. Higher frequency of diabetes mellitus in *KP *CAP patients was consistent with commonly reported association between diabetes condition and community-acquired *KP *bacteremia or community-acquired pleural effusion [24,25].

Hemoptysis is a clinical sign of interest in a TB endemic country. Hemoptysis is known to be associated with *KP *necrotizing pneumonia but is rarely described in the literature [26]. In our series, ~20% of the patients infected by *KP *had hemoptysis on admission. We failed to demonstrate an association between *KP *and hemoptysis as the frequency of TB and bronchiectasis--known as confounding factors--was high in our cohort *KP *accounted for 4.5% of documented pleural effusion. This number is lower than proportions found (12 to 23.6%) in other Asian countries [18,27,28]. As bacteriological analysis of pleural fluids was not available in our study, the proportion of *KP *pleural effusion might be underestimated.

Our finding also pointed to the fact many patients did not receive an appropriate antimicrobial treatment although the causal agent bundled with antibiotics susceptibility testing results was made available as soon as possible. Several reasons may explain this: (i) antibiotics susceptibility results were sent to the hospital while patients were already discharged (families usually request discharge when no hope of cure is expected), (ii) treatments may have been too expensive for some patients and their relatives, (iii) the prescription of penicillin A as a first line antibiotic in ALRI was the rule in these 2 hospitals but is known to be inefficient against *KP*. Another point is, the high prevalence of pulmonary sequelae, particularly TB sequelae in a highly endemic country, may be the cause of *KP *bronchiectasis colonization. In 50 Thaï patients diagnosed with bronchiectasis, *KP *was the second isolated bacterium (14%). Unfortunately, the authors did not report as whether this pathogen was found during an acute exacerbation or not [29].

Some limitations have to be underlined. The project was implemented for surveillance of ALRI related etiologies in settings where microbiology capacity was inexistent, although we were able to capture about all the patients who were admitted with acute respiratory symptoms, the frequency and the quality of specimens' collection for diagnosis was insufficient. Only ~50% of all included patients had available sputum (unpublished data). Capacity for more refined diagnosis was also limited by the absence of computed tomographic scan or capacity for aspiration by bronchial fibroscopy. Additionally, no colony forming unit/mL threshold was validated in patients with preexisting pulmonary lesions. In the context of surveillance, we did not request systematic verification of bacterial eradication at the end of the treatment, which made it difficult to confirm the causal implication of *KP *in the respiratory infection.

## Conclusions

Nevertheless, despite these limitations, our first documented series of *KP*-infected cases in Cambodia have shown particularly relevant information for Cambodian clinicians. As a result, we recommend a change of the current national approach in managing ALRI at the hospital level in the country. Antibiotics which include *KP *in their spectrum (i.e. amoxicillin/clavulanic acid, ceftriaxone) should be used when the first line antimicrobial therapy (mainly penicillin A in national Cambodian guidelines) fails or in case of necrotizing pneumonia. In any case, it is crucial for Cambodia to be able to acquire adequate microbiological capacity (i.e. culture) in public hospitals. Finally, the emergence or the rise of ESBL-producing strains causing community-acquired infections in Cambodia should draw the attention of the Cambodian health authorities as well as the international public health community.

## Competing interests

The authors declare that they have no competing interests.

## Authors' contributions

Conceived and designed the study: SV, BR, CM; Performed the study: BR, VT, PLT. Analyzed the data: BR, JB, SG. Contributed reagents/materials/analysis tools: BR, BG, SH, VT, PLT, CM, LB, PB, JB. Wrote the paper: BR, JB, SV. Critical review of the paper: All. Found funding: SV, PB. All authors read and approved the final manuscript.

## Pre-publication history

The pre-publication history for this paper can be accessed here:

http://www.biomedcentral.com/1471-2334/12/3/prepub

## References

[B1] Kang CI Kim SH Bang JW Kim HB Kim NJ Kim EC Oh MD Choe KW Community-acquired versus nosocomial Klebsiella pneumoniae bacteremia: clinical features, treatment outcomes, and clinical implication of antimicrobial resistance J Korean Med Sci 2006 21 5 816 822 10.3346/jkms.2006.21.5.816 17043412 PMC2721989

[B2] Paganin F Lilienthal F Bourdin A Lugagne N Tixier F Génin R Yvin JL Severe community-acquired pneumonia: assessment of microbial aetiology as mortality factor Eur Respir J 2004 24 5 779 785 10.1183/09031936.04.00119503 15516672

[B3] Bansal S Kashyap S Pal LS Goel A Clinical and bacteriological profile of community acquired pneumonia in Shimla, Himachal Pradesh Indian J Chest Dis Allied Sci 2004 46 1 17 22 14870864

[B4] Huang HH Zhang YY Xiu QY Zhou X Huang SG Lu Q Wang DM Wang F Community-acquired pneumonia in Shanghai, China: microbial etiology and implications for empirical therapy in a prospective study of 389 patients Eur J Clin Microbiol Infect Dis 2006 25 6 369 374 10.1007/s10096-006-0146-7 16767484

[B5] Reechaipichitkul W Lulitanond V Tantiwong P Saelee R Pisprasert V Etiologies and treatment outcomes in patients hospitalized with community-acquired pneumonia (CAP) at Srinagarind Hospital, Khon Kaen, Thailand Southeast Asian J Trop Med Public Health 2005 36 1 156 161 15906660

[B6] Ko WC Paterson DL Sagnimeni AJ Hansen DS Von Gottberg A Mohapatra S Casellas JM Goossens H Mulazimoglu L Trenholme G Klugman KP McCormack JG Yu VL Community-acquired Klebsiella pneumoniae bacteremia: global differences in clinical patterns Emerging Infect Dis 2002 8 2 160 166 10.3201/eid0802.010025 11897067 PMC2732457

[B7] Radyowijati A Haak H Improving antibiotic use in low-income countries: an overview of evidence on determinants Soc Sci Med 2003 57 4 733 744 10.1016/S0277-9536(02)00422-7 12821020

[B8] Song JH Oh WS Kang CI Chung DR Peck KR Ko KS Yeom JS Kim CK Kim SW Chang HH Kim YS Jung SI Tong Z Wang Q Huang SG Liu JW Lalitha MK Tan BH Van PH Carlos CC So T Asia Network for Surveillance of Resistant Pathogens Study Group Epidemiology and clinical outcomes of community-acquired pneumonia in adult patients in Asian countries: a prospective study by the Asian network for surveillance of resistant pathogens Int J Antimicrob Agents 2008 31 2 107 114 10.1016/j.ijantimicag.2007.09.014 18162378 PMC7134693

[B9] Hawkey PM Prevalence and clonality of extended-spectrum beta-lactamases in Asia Clin Microbiol Infect 2008 14 Suppl 1 159 165 18154540 10.1111/j.1469-0691.2007.01855.x

[B10] Turner PJ Extended-spectrum beta-lactamases Clin Infect Dis 2005 41 Suppl 4 S273 S275 16032564 10.1086/430789

[B11] Murray PR Washington JA Microscopic and bacteriologic analysis of expectorated sputum Mayo Clin Proc 1975 50 6 339 344 1127999

[B12] Lim WS van der Eerden MM Laing R Boersma WG Karalus N Town GI Lewis SA Macfarlane JT Defining community acquired pneumonia severity on presentation to hospital: an international derivation and validation study Thorax 2003 58 5 377 382 10.1136/thorax.58.5.377 12728155 PMC1746657

[B13] American Diabetes Association Postprandial blood glucose Diabetes Care 2001 24 4 775 778 11315848 10.2337/diacare.24.4.775

[B14] Franquet T Imaging of pneumonia: trends and algorithms Eur Respir J 2001 18 1 196 208 10.1183/09031936.01.00213501 11510793

[B15] Grenier P Image thoracique de l'adulte 1996 Paris: Flammarion Médecine-Science

[B16] Hsueh PR Badal RE Hawser SP Hoban DJ Bouchillon SK Ni Y Paterson DL Asia-Pacific SMART Group Epidemiology and antimicrobial susceptibility profiles of aerobic and facultative Gram-negative bacilli isolated from patients with intra-abdominal infections in the Asia-Pacific region: 2008 results from SMART (Study for Monitoring Antimicrobial Resistance Trends) Int J Antimicrob Agents 2010 36 5 408 414 2008 10.1016/j.ijantimicag.2010.07.002 20728316

[B17] Gorman S Pon D Sok K Gender and Development in Cambodia: An Overview http://www.cdri.org.kh/webdata/workpap/wp10-abs.htm

[B18] Mayor S Better access to drugs in developing countries is accelerating resistance BMJ 2010 340 c3234 10.1136/bmj.c3234 20558519

[B19] Ruppé E Hem S Lath S Gautier V Ariey F Sarthou JL Monchy D Arlet G CTX-M beta-lactamases in Escherichia coli from community-acquired urinary tract infections, Cambodia Emerging Infect Dis 2009 15 5 741 748 10.3201/eid1505.071299 19402960 PMC2687024

[B20] Lin YT Chen TL Siu LK Hsu SF Fung CP Clinical and microbiological characteristics of community-acquired thoracic empyema or complicated parapneumonic effusion caused by Klebsiella pneumoniae in Taiwan Eur J Clin Microbiol Infect Dis 2010 29 8 1003 1010 10.1007/s10096-010-0961-8 20505967

[B21] Loh LC Rosdara Masayuni MS Nor Izran Hanim AS Raman S Thayaparan T Kumar S Adverse hospital outcomes associated with the choice of empiric antibiotics in Klebsiella pneumoniae pneumonia: a retrospective observational study Ann Acad Med Singap 2007 36 8 642 646 17767334

[B22] Han XY Tarrand JJ Infante R Jacobson KL Truong M Clinical significance and epidemiologic analyses of Mycobacterium avium and Mycobacterium intracellulare among patients without AIDS J Clin Microbiol 2005 43 9 4407 4412 10.1128/JCM.43.9.4407-4412.2005 16145084 PMC1234053

[B23] Carey MA Card JW Voltz JW Germolec DR Korach KS Zeldin DC The impact of sex and sex hormones on lung physiology and disease: lessons from animal studies Am J Physiol Lung Cell Mol Physiol 2007 293 2 L272 L278 10.1152/ajplung.00174.2007 17575008

[B24] Lee C Liu J Su L Chien C Li C Yang K Hypermucoviscosity associated with Klebsiella pneumoniae-mediated invasive syndrome: a prospective cross-sectional study in Taiwan Int J Infect Dis 2010 14 8 e688 e692 10.1016/j.ijid.2010.01.007 20547084

[B25] Tsay RW Siu LK Fung CP Chang FY Characteristics of bacteremia between community-acquired and nosocomial Klebsiella pneumoniae infection: risk factor for mortality and the impact of capsular serotypes as a herald for community-acquired infection Arch Intern Med 2002 162 9 1021 1027 10.1001/archinte.162.9.1021 11996612

[B26] Prince SE Dominger KA Cunha BA Klein NC Klebsiella pneumoniae pneumonia Heart Lung 1997 26 5 413 417 10.1016/S0147-9563(97)90028-5 9315470

[B27] Lin Y Chen H Liu Y Shih C Hsu W Tu C A 30-month experience of thoracic empyema in a tertiary hospital: emphasis on differing bacteriology and outcome between the medical intensive care unit (MICU) and medical ward South Med J 2008 101 5 484 489 10.1097/SMJ.0b013e31816c00fa 18414163

[B28] Tsang KY Leung WS Chan VL Lin AWL Chu CM Complicated parapneumonic effusion and empyema thoracis: microbiology and predictors of adverse outcomes Hong Kong Med J 2007 13 3 178 186 17548905

[B29] Palwatwichai A Chaoprasong C Vattanathum A Wongsa A Jatakanon A Clinical, laboratory findings and microbiologic characterization of bronchiectasis in Thai patients Respirology 2002 7 1 63 66 10.1046/j.1440-1843.2002.00367.x 11896903

